# Prader-Willi Syndrome in Adulthood: A Case Report of Dermatologic and Ophthalmic Features Not Well Documented in the Literature

**DOI:** 10.7759/cureus.92962

**Published:** 2025-09-22

**Authors:** Isaac Elijah, Gurnoor S Gill, Allen Sklaver

**Affiliations:** 1 Medical School, Florida Atlantic University Charles E. Schmidt College of Medicine, Boca Raton, USA; 2 Internal Medicine, Florida Atlantic University Charles E. Schmidt College of Medicine, Boca Raton, USA

**Keywords:** chronic systemic inflammation, genetic syndromes, prader-willi syndrome, psoriasis, retinal hypopigmentation

## Abstract

Prader-Willi syndrome (PWS) is a rare genetic disorder that presents in infancy with feeding difficulties and hypotonia, later progressing to compulsive eating and metabolic complications. This case describes a 32-year-old male with genetically undiagnosed PWS, presenting with progressive muscular weakness, severe obesity (BMI 39.3), cognitive impairment, and functional decline. Notably, he exhibited psoriasis and ophthalmic abnormalities (bilateral proptosis and a dense cataract), which are not commonly associated with PWS. These findings reflect potential secondary complications from prolonged metabolic dysfunction and inadequate medical management into adulthood. This case highlights the severe consequences of late diagnosis, including uncontrolled obesity, hypertension, prediabetes, and musculoskeletal impairment. The presence of psoriasis and ocular findings suggests a potential interplay between chronic inflammation, metabolic syndrome, and PWS-related endocrine dysfunction. This case highlights the potential importance of early recognition, multidisciplinary management, and long-term follow-up in individuals with PWS, which may help reduce the risk of progressive disability and associated comorbidities.

## Introduction

Prader-Willi Syndrome (PWS) is a rare genetic disorder caused by the loss of paternal gene expression at chromosome 15q11-q13, typically due to a paternal deletion, maternal uniparental disomy (UPD), or an imprinting defect [1]. It is a complex neurodevelopmental and metabolic disorder characterized by hypotonia, hyperphagia, severe obesity, intellectual disability, endocrine dysfunction, and behavioral abnormalities [2]. The syndrome follows a distinct clinical trajectory, with neonatal hypotonia and feeding difficulties, progressing to insatiable appetite and excessive weight gain in early childhood, often resulting in severe obesity if not strictly managed. However, many individuals remain undiagnosed until adulthood, as the early features may be overlooked and the later manifestations (such as behavioral issues, metabolic syndrome, or musculoskeletal decline) are nonspecific and can mimic other conditions, complicating timely recognition [3].

Patients with PWS frequently experience short stature, growth hormone deficiency (GHD), hypogonadism, and hypothyroidism, contributing to delayed puberty, osteoporosis, and metabolic complications such as type 2 diabetes mellitus (T2DM), hypertension (HTN), and cardiovascular disease (CVD) [4]. Cognitive impairment is nearly universal, typically manifesting as mild to moderate intellectual disability, delayed language acquisition, and deficits in executive function and social interactions [5]. Additionally, behavioral dysregulation, including obsessive-compulsive tendencies, emotional outbursts, skin picking, and rigid thought patterns, is common and often complicates management [6].

While PWS is associated with characteristic craniofacial features (narrow forehead, almond-shaped eyes, thin upper lip, and a downturned mouth) and musculoskeletal abnormalities (hypotonia, scoliosis, and joint laxity), this case is particularly notable for the presence of psoriasis and ophthalmic abnormalities, including bilateral proptosis and a dense cataract, which are not typically seen in PWS [7]. These findings may be secondary complications from prolonged metabolic dysfunction, systemic inflammation, or inadequate medical management into adulthood [8]. Given the complexity of PWS and the high risk of multisystem involvement, early diagnosis, intensive dietary control, endocrinologic intervention, and long-term multidisciplinary management are crucial to mitigating complications and improving patient outcomes [9]. This case underscores the importance of early recognition and intervention in PWS to prevent progressive metabolic and functional decline.

## Case presentation

A 32-year-old male from Colombia was first seen on May 24, 2024, for evaluation. His primary complaint was progressive muscular weakness over the past five years, which had worsened to the point where he had difficulty standing without assistance. The patient’s mother reported that he had previously been given a presumptive diagnosis of PWS; however, no genetic confirmation or medical documentation was available, and he had not pursued further formal evaluation. Genetic testing was not available in this case because of factors such as resource limitations and patient-specific circumstances. The diagnosis was therefore made on clinical grounds, supported by characteristic features and multidisciplinary consensus, though we acknowledge that molecular confirmation is the diagnostic gold standard. In addition to muscular weakness, he reported persistent hunger and obesity. He reported loss of vision in his left eye. The etiology was presumed to be cataract and myopia, though trauma could not be definitively excluded in the absence of prior records. Formal assessment of visual acuity at presentation is described below. His vaccination records were lost.

His family history was notable for a maternal grandmother with diabetes mellitus. His mother and two siblings were alive and well. He had no history of smoking or alcohol use. His vaccination records were unavailable. On physical examination, his height was 4 feet 7 inches, his weight was 172 pounds, and his BMI was 39.3, classifying him as obese. His blood pressure was 142/89 mmHg, and his oxygen saturation was 100% on room air. On ophthalmologic evaluation, he exhibited severe myopia and a dense cataract in the left eye, with markedly reduced visual acuity consistent with functional blindness by WHO criteria [10]. Formal refraction and intraocular pressure measurements were not available at the time of evaluation, which is a limitation of this report. Patient presented with plaques on his forehead and bilateral knees and elbows, consistent with psoriasis as confirmed by clinical examination.

The patient exhibited characteristic craniofacial features of PWS, including a thin upper lip, narrow nasal bridge, and narrow forehead. Neurologic and musculoskeletal examination revealed bilateral external foot deviation (he was noted to wear his right shoe on the left foot, and vice versa) along with reduced muscle strength in both upper and lower extremities (3/5). While the diagnosis of PWS is ideally confirmed through molecular genetic testing, such testing was not feasible in this case due to the patient’s uninsured status and limited resources. The patient was evaluated at the Caridad Clinic, a nonprofit community clinic in South Florida. where access to advanced diagnostics, such as DNA methylation analysis, was unavailable. Despite this limitation, the combination of his craniofacial morphology, muscular hypotonia, endocrinologic features, obesity, and behavioral history strongly supported a presumptive clinical diagnosis of PWS.

He had significant difficulty standing without assistance. Cognitively, he was alert and oriented and able to state his name, but he displayed marked intellectual disability. His laboratory findings can be seen in Table 1.

**Table 1:** Patient's laboratory findings HbA1c: glycated hemoglobin; WBC: white blood cells; TSH: thyroid-stimulating hormone; T4: thyroxine; HDL: high-density lipoprotein; LDL: low-density lipoprotein; Chol/HDL ratio: cholesterol to high-density lipoprotein ratio

Test	Result	Reference range
HbA1c	6.2%	< 5.7% (normal), 5.7-6.4% (prediabetes), ≥6.5% (diabetes)
WBC	10.4 x10^9/L	4.0-11.0 x10^9/L
Hemoglobin	13.5 g/dL	13.2-16.6 g/dL
Hematocrit	41%	38-50%
Platelets	317,000/mm³	150,000-450,000/mm³
TSH	1.35 µIU/mL	0.5-4.7 µIU/mL
T4	1.1 µg/dL	0.8-1.8 µg/dL
Total cholesterol	187 mg/dL	< 200 mg/dL
HDL	41 mg/dL	> 40 mg/dL
Triglycerides	193 mg/dL	< 150 mg/dL
LDL	115 mg/dL	< 100 mg/dL (optimal)
Chol/HDL ratio	4.6	< 5.0

The patient’s triglycerides were elevated at 193 mg/dL (reference <150 mg/dL), consistent with borderline hypertriglyceridemia, which may reflect underlying metabolic dysfunction associated with obesity and possible insulin resistance. The patient’s LDL cholesterol was above the optimal range (115 mg/dL), which, in combination with elevated triglycerides and obesity, reflects an atherogenic dyslipidemia. Such lipid abnormalities are common in PWS and contribute to heightened cardiovascular risk through accelerated atherosclerosis, particularly in the context of insulin resistance and metabolic syndrome.

The patient was prescribed a 17% salicylic acid and 0.05% clobetasol topical ointment for the management of psoriasis. On ophthalmology follow-up eight weeks later, examination confirmed bilateral proptosis with intraocular pressures within normal limits and a mature cataract in the left eye, accompanied by 360-degree posterior synechiae to the iris. This was the only complete ophthalmologic evaluation available for this patient, and therefore, earlier descriptions of his vision are limited to clinical observations rather than formal measurements. Thyroid autoantibodies (TSI, TPO) were not available at our clinic, but thyroid function tests (TSH and T4) were within normal limits, making thyroid eye disease a less likely etiology of the proptosis.

## Discussion

PWS manifests as a heterogeneous array of clinical features that evolve across the lifespan. Although the clinical features of PWS evolve over time, certain manifestations such as hyperphagia and obesity risk often persist into adulthood. This persistence is explained by the underlying hypothalamic dysfunction characteristic of PWS, which leads to impaired satiety signaling and abnormal regulation of appetite throughout life. While neonatal hypotonia and poor feeding improve with age, the subsequent phase of hyperphagia emerges in early childhood and typically remains chronic [1]. Without strict dietary control and multidisciplinary management, these feeding-related issues continue to drive obesity, metabolic syndrome, and associated comorbidities well into adulthood, as observed in our patient [2,3].

Hallmark manifestations include neonatal hypotonia, hyperphagia, intellectual disability, and endocrine dysfunction, with additional neurobehavioral and musculoskeletal complications well described in the literature [1,2]. To our knowledge, the coexistence of psoriasis and bilateral proptosis in PWS has not previously been well documented. Importantly, recent studies have suggested that individuals with PWS may exhibit a state of chronic low-grade inflammation, reflected by elevated C-reactive protein, interleukin-6, and other inflammatory markers. This case highlights the potential interaction between such inflammatory pathways and dermatologic/ocular comorbidities, suggesting that chronic inflammation could act as a pathophysiological contributor, that is, a biological mechanism that amplifies or sustains disease processes beyond the classical genetic and endocrine abnormalities of PWS. In this context, inflammation may compound the syndrome’s metabolic dysregulation and increase susceptibility to conditions such as psoriasis and possibly ocular abnormalities, broadening the underrecognized clinical spectrum of PWS [11].

This hypothesis aligns with prior studies identifying heightened low-grade systemic inflammation in PWS independent of obesity. Marelli et al. described a case of PWS with inflammatory arthritis and anterior uveitis, suggesting that the concomitant presence of the predefined genetic syndrome could play a role in the development of joint inflammation [12]. Caixas et al. found that PWS subjects displayed additional low-grade systemic inflammation compared to age and sex-matched obese controls, supporting the notion of an underlying pathophysiological connection to inflammatory states that goes beyond the metabolic syndrome characteristic of PWS [13]. While the diagnosis of PWS is most accurately confirmed through genetic testing (e.g., DNA methylation analysis or FISH for chromosome 15q11-q13 abnormalities), this was not performed in our patient due to limited resources and lack of insurance coverage at the Caridad Clinic. Instead, the diagnosis was made on strong clinical grounds, given the presence of hallmark craniofacial, metabolic, musculoskeletal, and behavioral features consistent with PWS. Additionally, although no inflammatory markers such as C-reactive protein (CRP) or erythrocyte sedimentation rate (ESR) were obtained, the dermatologic findings (psoriasis with extensive plaques) and metabolic abnormalities raise the possibility of an underlying pro-inflammatory state. This limitation underscores the need for further investigation into the role of chronic inflammation in PWS, especially in resource-limited settings where genetic and advanced laboratory testing may not be feasible

Psoriasis, a chronic, immune-mediated inflammatory skin disorder, is increasingly recognized as a systemic disease with significant associations with obesity, metabolic syndrome, and cardiovascular comorbidities - features commonly observed in PWS. Armstrong et al. demonstrated a significant relationship between psoriasis and obesity in a meta-analysis of observational studies, identifying shared pro-inflammatory cytokine pathways, particularly involving tumor necrosis factor-alpha (TNF-α) and interleukin-6 (IL-6), which may exacerbate the metabolic dysfunction characteristic of PWS [14]. Since psoriasis is a chronic immune-mediated inflammatory condition driven largely by dysregulated cytokine pathways, including TNF-α and IL-6, these same cytokines have been implicated in worsening metabolic dysfunction, insulin resistance, and systemic inflammation [12-13]. In PWS, hypothalamic dysfunction predisposes individuals to obesity, metabolic syndrome, and endocrine abnormalities, which themselves promote a pro-inflammatory state. When combined, psoriasis and PWS may act synergistically to amplify systemic inflammation through a positive feedback loop: metabolic derangements in PWS increase circulating inflammatory cytokines, while psoriasis perpetuates cytokine release, further worsening insulin resistance and metabolic dysfunction [1-3,12]. This bidirectional interaction may help explain how dermatologic manifestations and metabolic features coexist and potentially exacerbate one another in our patient. Furthermore, Mrowietz et al. have emphasized the systemic nature of psoriasis, reporting its interplay with metabolic syndrome and inflammatory states in predisposed individuals [14-17]. These findings suggest that psoriasis in this patient may reflect an inflammatory amplification loop, wherein the endocrine and metabolic derangements of PWS perpetuate systemic inflammation, thereby predisposing to dermatologic manifestations. 

The patient, in this case, had an extensive history of myopia and cataracts in the left eye, lateral deviation of the left eye, and bilateral proptosis. Previous reports have described various ocular pathologies in PWS, including telecanthus, strabismus, nystagmus, foveal hypoplasia, visual field defects, cataracts, and retinal hypopigmentation [15]. Bilateral proptosis has not been previously associated with PWS and is likely unrelated to underlying autoimmune thyroiditis in the present case, given the unremarkable thyroid panel. Wang et al. have described associations between systemic diseases and ocular pathology, noting that metabolic and endocrine dysfunctions frequently present with diverse ocular manifestations, including cataract formation and myopia, due to alterations in glucose metabolism, lipid profiles, and growth factor levels [16]. The bilateral proptosis observed in this patient may not be solely attributable to PWS, as proptosis has not been consistently described in the syndrome. Alternative causes should also be considered. Inflammatory conditions such as thyroid eye disease (Graves’ orbitopathy) can present with bilateral proptosis, though this was unlikely in our patient given a normal thyroid panel [10]. Structural etiologies, including orbital masses, vascular malformations (e.g., carotid-cavernous fistula, orbital varices), and congenital craniofacial anomalies, are also important differentials. Additionally, chronic systemic inflammation associated with psoriasis and metabolic dysfunction in PWS could contribute indirectly to orbital tissue remodeling and periocular fat expansion, potentially compounding the clinical picture. While the absence of thyroid dysfunction and orbital imaging limits definitive exclusion of other causes, the coexistence of psoriasis and metabolic derangements supports the hypothesis of an inflammatory contribution to the ocular findings in this patient [17-19].

Additionally, the broader dermatologic burden associated with systemic diseases supports this inflammatory dynamic. Karimkhani et al. quantified the substantial morbidity associated with skin diseases in systemic conditions, reinforcing the need to recognize cutaneous signs as markers of underlying multisystem pathology [15,20]. Given the inflammatory overlap between psoriasis, obesity, and metabolic dysfunction, dermatologic manifestations in PWS may be underreported or misattributed for several reasons [1-5]. First, skin findings such as plaques, rashes, or xerosis may be mistakenly attributed to poor hygiene, obesity-related skin changes (e.g., intertrigo, acanthosis nigricans), or secondary infections, rather than recognized as primary inflammatory dermatoses. Second, patients with PWS often have communication and cognitive challenges, limiting their ability to report pruritus or evolving cutaneous symptoms, which delays recognition. Third, in low-resource settings, limited access to dermatology further increases the risk of misdiagnosis or underreporting. These factors underscore the need for heightened dermatologic surveillance in PWS, as skin manifestations may serve as clinically important markers of systemic inflammation and metabolic dysregulation. A summary of previously reported ophthalmic and dermatologic manifestations in PWS, contextualized alongside the present case, is provided in Table 2 to highlight the rarity and heterogeneity of these findings [21-25].

**Table 2:** Reported ophthalmic and dermatologic case findings in PWS OCT: optical coherence tomography; PWS: Prader-Willi syndrome

Author	Patient	Manifestation	Relevance to current case
Hamid et al. [21]	30-y male	Absent foveal pit (OCT), macular neovascularization	Demonstrates rare retinal pathology in adult PWS, parallels unusual ocular findings in our case (proptosis, cataract)
Marelli et al. [12]	2 children	Inflammatory arthritis, one with chronic bilateral anterior uveitis	Shows systemic inflammatory overlap and ophthalmic inflammation in PWS, relevant to bilateral ocular disease in our case
Futterweit et al. [22]	Child	Congenital ectropion uveae + glaucoma	Rare developmental ocular anomaly in PWS, underscores ocular diversity
Wang et al. [16]	25-y woman	Retinal hypopigmentation, lens changes	Adds to spectrum of structural ocular abnormalities; parallels cataract in our case
Hall et al. [23]	Teen	Severe self-injurious skin-picking	Highlights dermatologic/behavioral overlap; contrasts with psoriasis in our case
Fowler & Butler [24]	Child	Urticaria pigmentosa (mastocytosis)	Early report of cutaneous self-injury, relevant for dermatology spectrum in PWS
Bornhausen-Demarch et al. [25]	22-y female	Dermatologic manifestations (seborrheic dermatitis, recurrent infections)	Illustrates variability in dermatologic disease expression in PWS

## Conclusions

This case underscores the complexity of PWS, highlighting how atypical manifestations such as psoriasis and bilateral proptosis may intersect with the syndrome’s well-recognized metabolic and endocrine abnormalities. Recognition of these unusual features is important, as they may serve as early clinical markers for multisystem involvement and warrant timely, multidisciplinary management. Moving forward, investigations should be conducted in a systematic manner-beginning with a confirmed genetic diagnosis of PWS to establish diagnostic certainty-followed by evaluation of inflammatory and metabolic pathways that may contribute to the broader morbidity profile of affected individuals. Such an approach not only refines diagnostic accuracy but also lays the groundwork for more targeted screening and management strategies, particularly in adult patients and those in low-resource settings where access to genetic testing remains limited.
